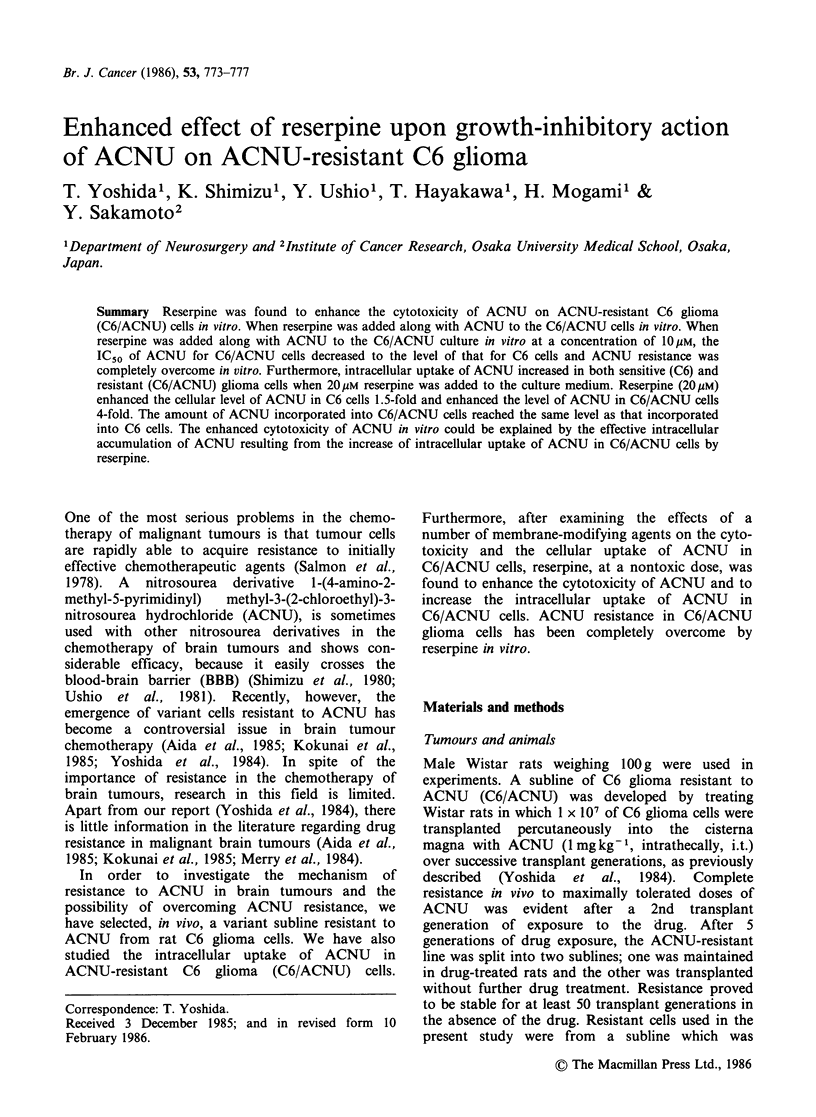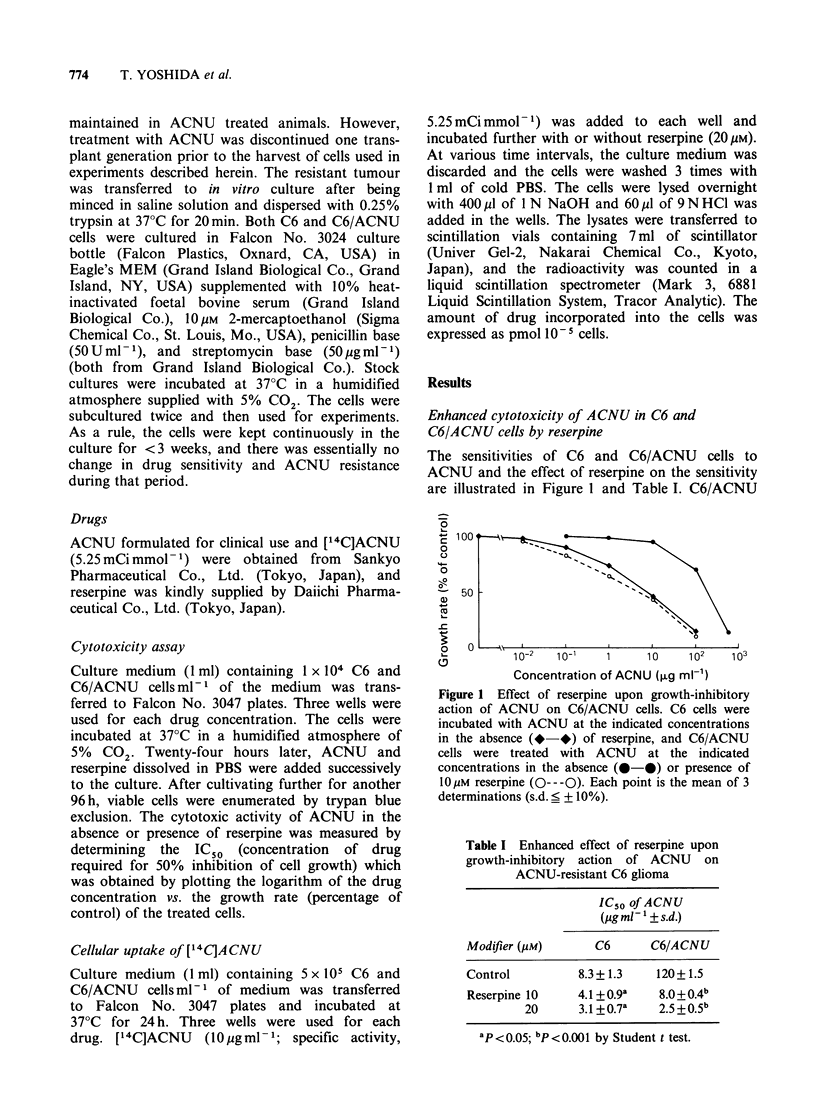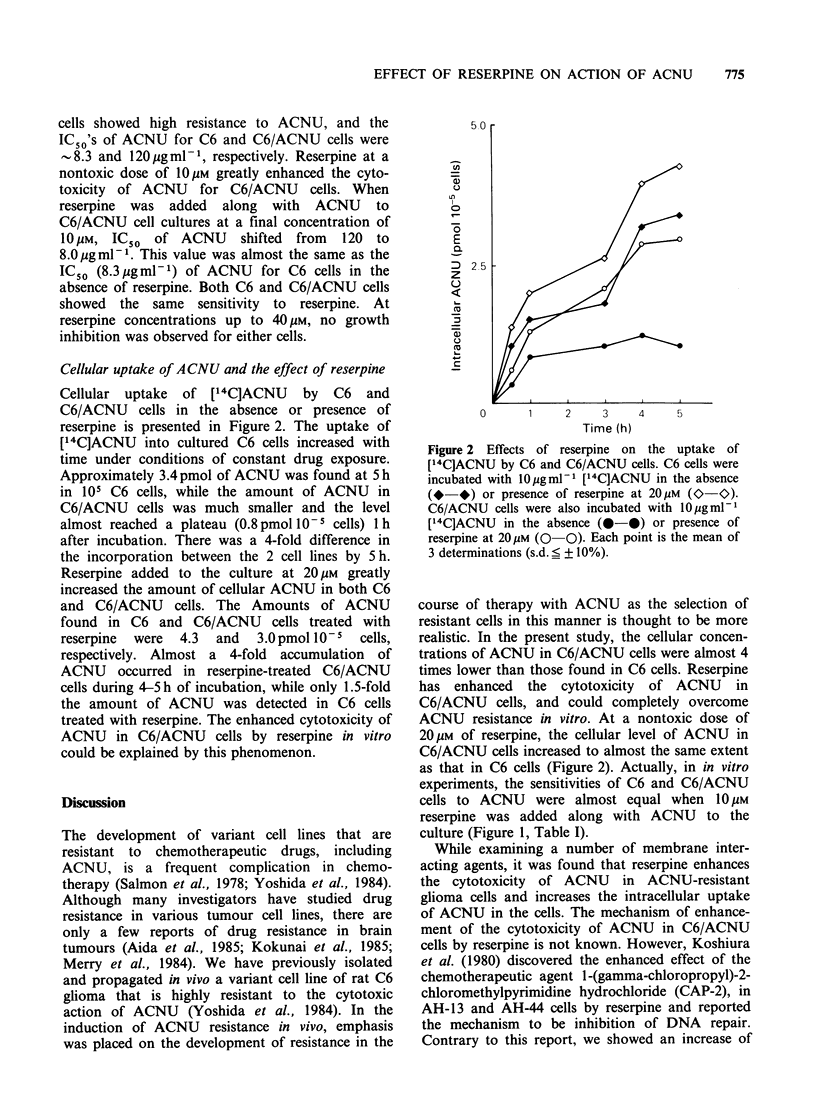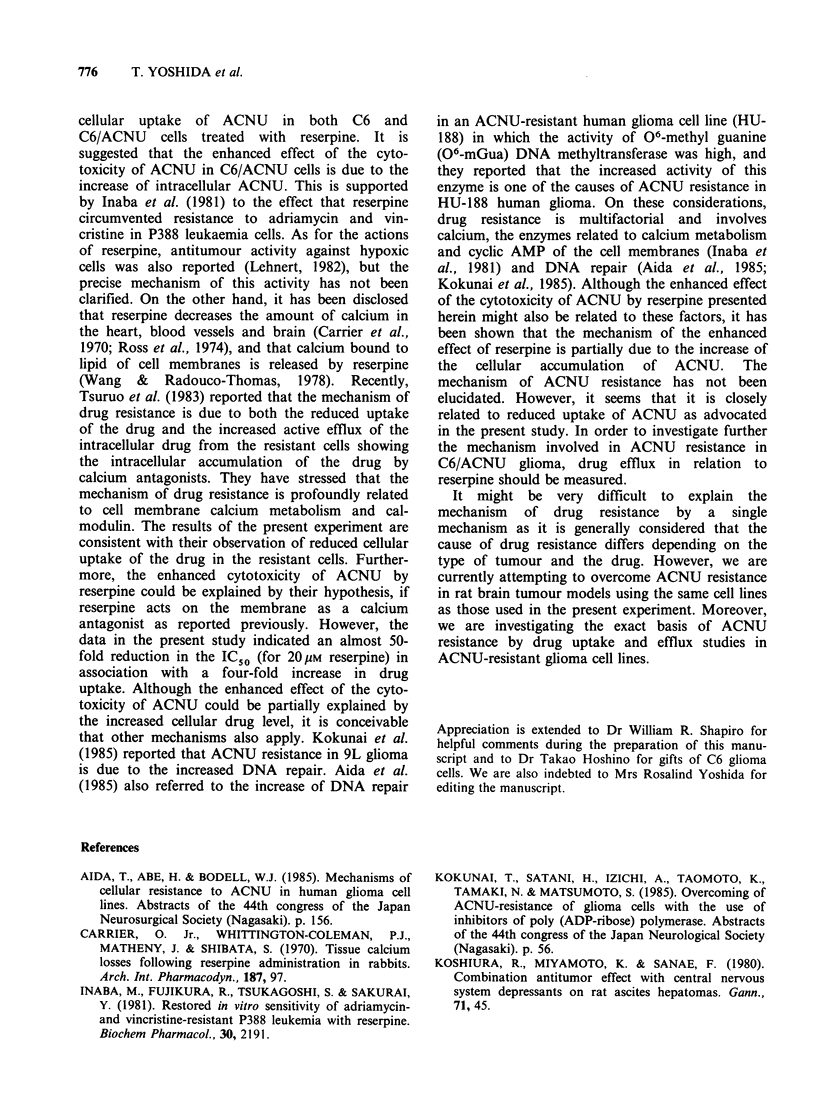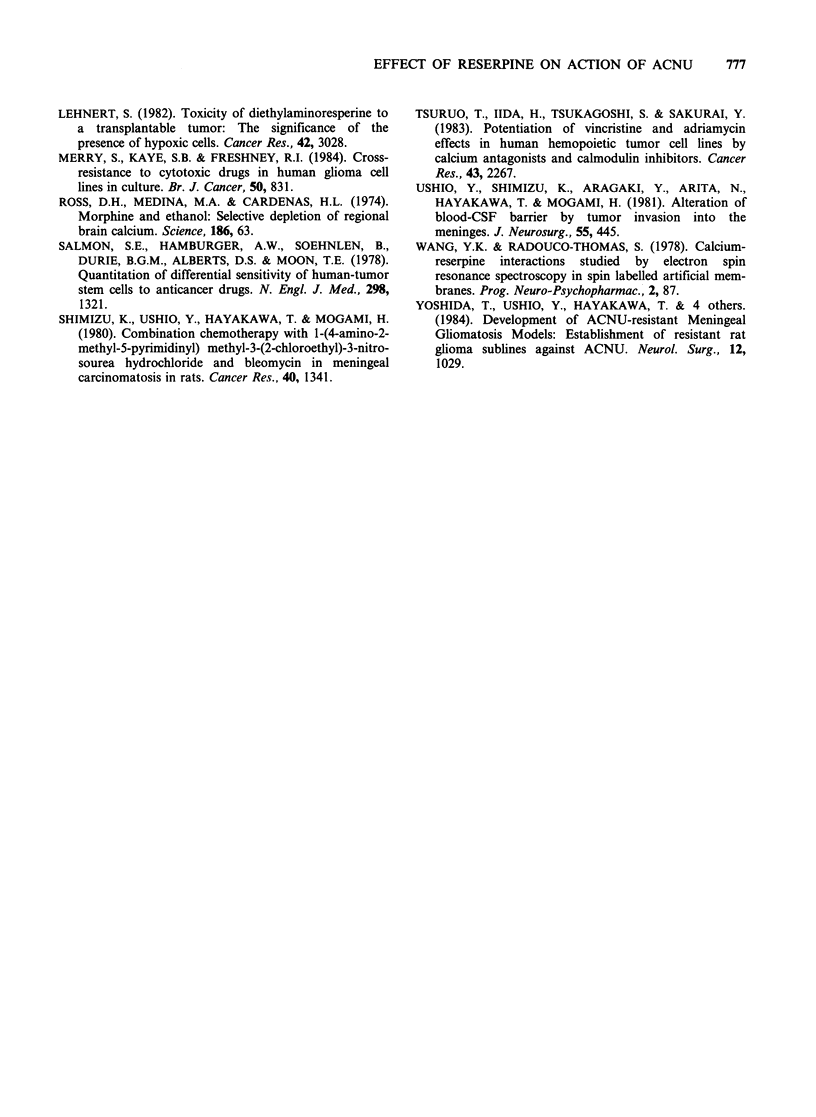# Enhanced effect of reserpine upon growth-inhibitory action of ACNU on ACNU-resistant C6 glioma.

**DOI:** 10.1038/bjc.1986.131

**Published:** 1986-06

**Authors:** T. Yoshida, K. Shimizu, Y. Ushio, T. Hayakawa, H. Mogami, Y. Sakamoto

## Abstract

Reserpine was found to enhance the cytotoxicity of ACNU on ACNU-resistant C6 glioma (C6/ACNU) cells in vitro. When reserpine was added along with ACNU to the C6/ACNU cells in vitro. When reserpine was added along with ACNU to the C6/ACNU culture in vitro at a concentration of 10 microM, the IC50 of ACNU for C6/ACNU cells decreased to the level of that for C6 cells and ACNU resistance was completely overcome in vitro. Furthermore, intracellular uptake of ACNU increased in both sensitive (C6) and resistant (C6/ACNU) glioma cells when 20 microM reserpine was added to the culture medium. Reserpine (20 microM) enhanced the cellular level of ACNU in C6 cells 1.5-fold and enhanced the level of ACNU in C6/ACNU cells 4-fold. The amount of ACNU incorporated into C6/ACNU cells reached the same level as that incorporated into C6 cells. The enhanced cytotoxicity of ACNU in vitro could be explained by the effective intracellular accumulation of ACNU resulting from the increase of intracellular uptake of ACNU in C6/ACNU cells by reserpine.


*Br. J. Cancer* (1986), **53**, 773–777

### Enhanced effect of reserpine upon growth-inhibitory action of ACNU on ACNU-resistant C6 glioma

T. Yoshida[1], K. Shimizu[1], Y. Ushio[1], T. Hayakawa[1], H. Mogami[1] & Y. Sakamoto[2]

[1]*Department of Neurosurgery and* [2]*Institute of Cancer Research, Osaka University Medical School, Osaka, Japan.*

**Summary** Reserpine was found to enhance the cytotoxicity of ACNU on ACNU-resistant C6 glioma (C6/ACNU) cells *in vitro*. When reserpine was added along with ACNU to the C6/ACNU cells *in vitro*. When reserpine was added along with ACNU to the C6/ACNU culture *in vitro* at a concentration of 10 $\mu$M, the $IC_{50}$ of ACNU for C6/ACNU cells decreased to the level of that for C6 cells and ACNU resistance was completely overcome *in vitro*. Furthermore, intracellular uptake of ACNU increased in both sensitive (C6) and resistant (C6/ACNU) glioma cells when 20 $\mu$M reserpine was added to the culture medium. Reserpine (20 $\mu$M) enhanced the cellular level of ACNU in C6 cells 1.5-fold and enhanced the level of ACNU in C6/ACNU cells 4-fold. The amount of ACNU incorporated into C6/ACNU cells reached the same level as that incorporated into C6 cells. The enhanced cytotoxicity of ACNU *in vitro* could be explained by the effective intracellular accumulation of ACNU resulting from the increase of intracellular uptake of ACNU in C6/ACNU cells by reserpine.

One of the most serious problems in the chemotherapy of malignant tumours is that tumour cells are rapidly able to acquire resistance to initially effective chemotherapeutic agents (Salmon *et al.*, 1978). A nitrosourea derivative 1-(4-amino-2-methyl-5-pyrimidinyl) methyl-3-(2-chloroethyl)-3-nitrosourea hydrochloride (ACNU), is sometimes used with other nitrosourea derivatives in the chemotherapy of brain tumours and shows considerable efficacy, because it easily crosses the blood-brain barrier (BBB) (Shimizu *et al.*, 1980; Ushio *et al.*, 1981). Recently, however, the emergence of variant cells resistant to ACNU has become a controversial issue in brain tumour chemotherapy (Aida *et al.*, 1985; Kokunai *et al.*, 1985; Yoshida *et al.*, 1984). In spite of the importance of resistance in the chemotherapy of brain tumours, research in this field is limited. Apart from our report (Yoshida *et al.*, 1984), there is little information in the literature regarding drug resistance in malignant brain tumours (Aida *et al.*, 1985; Kokunai *et al.*, 1985; Merry *et al.*, 1984).

In order to investigate the mechanism of resistance to ACNU in brain tumours and the possibility of overcoming ACNU resistance, we have selected, *in vivo*, a variant subline resistant to ACNU from rat C6 glioma cells. We have also studied the intracellular uptake of ACNU in ACNU-resistant C6 glioma (C6/ACNU) cells. Furthermore, after examining the effects of a number of membrane-modifying agents on the cytotoxicity and the cellular uptake of ACNU in C6/ACNU cells, reserpine, at a nontoxic dose, was found to enhance the cytotoxicity of ACNU and to increase the intracellular uptake of ACNU in C6/ACNU cells. ACNU resistance in C6/ACNU glioma cells has been completely overcome by reserpine *in vitro*.

Correspondence: T. Yoshida.
Received 3 December 1985; and in revised form 10 February 1986.

### Materials and methods

### *Tumours and animals*

Male Wistar rats weighing 100 g were used in experiments. A subline of C6 glioma resistant to ACNU (C6/ACNU) was developed by treating Wistar rats in which $1 \times 10^7$ of C6 glioma cells were transplanted percutaneously into the cisterna magna with ACNU (1 mg kg$^{-1}$, intrathecally, i.t.) over successive transplant generations, as previously described (Yoshida *et al.*, 1984). Complete resistance *in vivo* to maximally tolerated doses of ACNU was evident after a 2nd transplant generation of exposure to the drug. After 5 generations of drug exposure, the ACNU-resistant line was split into two sublines; one was maintained in drug-treated rats and the other was transplanted without further drug treatment. Resistance proved to be stable for at least 50 transplant generations in the absence of the drug. Resistant cells used in the present study were from a subline which was

© The Macmillan Press Ltd., 1986

maintained in ACNU treated animals. However, treatment with ACNU was discontinued one transplant generation prior to the harvest of cells used in experiments described herein. The resistant tumour was transferred to *in vitro* culture after being minced in saline solution and dispersed with 0.25% trypsin at 37°C for 20 min. Both C6 and C6/ACNU cells were cultured in Falcon No. 3024 culture bottle (Falcon Plastics, Oxnard, CA, USA) in Eagle's MEM (Grand Island Biological Co., Grand Island, NY, USA) supplemented with 10% heat-inactivated foetal bovine serum (Grand Island Biological Co.), 10 μM 2-mercaptoethanol (Sigma Chemical Co., St. Louis, Mo., USA), penicillin base (50 U $ml^{-1}$), and streptomycin base (50 μg $ml^{-1}$) (both from Grand Island Biological Co.). Stock cultures were incubated at 37°C in a humidified atmosphere supplied with 5% $CO_2$. The cells were subcultured twice and then used for experiments. As a rule, the cells were kept continuously in the culture for <3 weeks, and there was essentially no change in drug sensitivity and ACNU resistance during that period.

### *Drugs*

ACNU formulated for clinical use and [$^{14}$C]ACNU (5.25 mCi $mmol^{-1}$) were obtained from Sankyo Pharmaceutical Co., Ltd. (Tokyo, Japan), and reserpine was kindly supplied by Daiichi Pharmaceutical Co., Ltd. (Tokyo, Japan).

### *Cytotoxicity assay*

Culture medium (1 ml) containing $1 \times 10^4$ C6 and C6/ACNU cells $ml^{-1}$ of the medium was transferred to Falcon No. 3047 plates. Three wells were used for each drug concentration. The cells were incubated at 37°C in a humidified atmosphere of 5% $CO_2$. Twenty-four hours later, ACNU and reserpine dissolved in PBS were added successively to the culture. After cultivating further for another 96 h, viable cells were enumerated by trypan blue exclusion. The cytotoxic activity of ACNU in the absence or presence of reserpine was measured by determining the $IC_{50}$ (concentration of drug required for 50% inhibition of cell growth) which was obtained by plotting the logarithm of the drug concentration *vs.* the growth rate (percentage of control) of the treated cells.

### *Cellular uptake of* [$^{14}$C]*ACNU*

Culture medium (1 ml) containing $5 \times 10^5$ C6 and C6/ACNU cells $ml^{-1}$ of medium was transferred to Falcon No. 3047 plates and incubated at 37°C for 24 h. Three wells were used for each drug. [$^{14}$C]ACNU (10 μg $ml^{-1}$; specific activity, 5.25 mCi $mmol^{-1}$) was added to each well and incubated further with or without reserpine (20 μM). At various time intervals, the culture medium was discarded and the cells were washed 3 times with 1 ml of cold PBS. The cells were lysed overnight with 400 μl of 1 N NaOH and 60 μl of 9 N HCl was added in the wells. The lysates were transferred to scintillation vials containing 7 ml of scintillator (Univer Gel-2, Nakarai Chemical Co., Kyoto, Japan), and the radioactivity was counted in a liquid scintillation spectrometer (Mark 3, 6881 Liquid Scintillation System, Tracor Analytic). The amount of drug incorporated into the cells was expressed as pmol $10^{-5}$ cells.

### Results

### *Enhanced cytotoxicity of ACNU in C6 and C6/ACNU cells by reserpine*

The sensitivities of C6 and C6/ACNU cells to ACNU and the effect of reserpine on the sensitivity are illustrated in Figure 1 and Table I. C6/ACNU

**Figure 1** Effect of reserpine upon growth-inhibitory action of ACNU on C6/ACNU cells. C6 cells were incubated with ACNU at the indicated concentrations in the absence (◆—◆) of reserpine, and C6/ACNU cells were treated with ACNU at the indicated concentrations in the absence (●—●) or presence of 10 μM reserpine (○---○). Each point is the mean of 3 determinations (s.d. ≦ ±10%).

**Table I** Enhanced effect of reserpine upon growth-inhibitory action of ACNU on ACNU-resistant C6 glioma

| | *$IC_{50}$ of ACNU (μg $ml^{-1}$ ± s.d.)* | |
|---|---|---|
| *Modifier (μM)* | *C6* | *C6/ACNU* |
| Control | 8.3 ± 1.3 | 120 ± 1.5 |
| Reserpine 10 | 4.1 ± 0.9[a] | 8.0 ± 0.4[b] |
| 20 | 3.1 ± 0.7[a] | 2.5 ± 0.5[b] |

[a]$P < 0.05$; [b]$P < 0.001$ by Student *t* test.

cells showed high resistance to ACNU, and the $IC_{50}$'s of ACNU for C6 and C6/ACNU cells were ~8.3 and 120 μg ml$^{-1}$, respectively. Reserpine at a nontoxic dose of 10 μM greatly enhanced the cytotoxicity of ACNU for C6/ACNU cells. When reserpine was added along with ACNU to C6/ACNU cell cultures at a final concentration of 10 μM, $IC_{50}$ of ACNU shifted from 120 to 8.0 μg ml$^{-1}$. This value was almost the same as the $IC_{50}$ (8.3 μg ml$^{-1}$) of ACNU for C6 cells in the absence of reserpine. Both C6 and C6/ACNU cells showed the same sensitivity to reserpine. At reserpine concentrations up to 40 μM, no growth inhibition was observed for either cells.

*Cellular uptake of ACNU and the effect of reserpine*

Cellular uptake of [$^{14}$C]ACNU by C6 and C6/ACNU cells in the absence or presence of reserpine is presented in Figure 2. The uptake of [$^{14}$C]ACNU into cultured C6 cells increased with time under conditions of constant drug exposure. Approximately 3.4 pmol of ACNU was found at 5 h in $10^5$ C6 cells, while the amount of ACNU in C6/ACNU cells was much smaller and the level almost reached a plateau (0.8 pmol $10^{-5}$ cells) 1 h after incubation. There was a 4-fold difference in the incorporation between the 2 cell lines by 5 h. Reserpine added to the culture at 20 μM greatly increased the amount of cellular ACNU in both C6 and C6/ACNU cells. The Amounts of ACNU found in C6 and C6/ACNU cells treated with reserpine were 4.3 and 3.0 pmol $10^{-5}$ cells, respectively. Almost a 4-fold accumulation of ACNU occurred in reserpine-treated C6/ACNU cells during 4–5 h of incubation, while only 1.5-fold the amount of ACNU was detected in C6 cells treated with reserpine. The enhanced cytotoxicity of ACNU in C6/ACNU cells by reserpine *in vitro* could be explained by this phenomenon.

**Figure 2** Effects of reserpine on the uptake of [$^{14}$C]ACNU by C6 and C6/ACNU cells. C6 cells were incubated with 10 μg ml$^{-1}$ [$^{14}$C]ACNU in the absence (◆—◆) or presence of reserpine at 20 μM (◇—◇). C6/ACNU cells were also incubated with 10 μg ml$^{-1}$ [$^{14}$C]ACNU in the absence (●—●) or presence of reserpine at 20 μM (○—○). Each point is the mean of 3 determinations (s.d. ≦ ±10%).

### Discussion

The development of variant cell lines that are resistant to chemotherapeutic drugs, including ACNU, is a frequent complication in chemotherapy (Salmon *et al.*, 1978; Yoshida *et al.*, 1984). Although many investigators have studied drug resistance in various tumour cell lines, there are only a few reports of drug resistance in brain tumours (Aida *et al.*, 1985; Kokunai *et al.*, 1985; Merry *et al.*, 1984). We have previously isolated and propagated *in vivo* a variant cell line of rat C6 glioma that is highly resistant to the cytotoxic action of ACNU (Yoshida *et al.*, 1984). In the induction of ACNU resistance *in vivo*, emphasis was placed on the development of resistance in the course of therapy with ACNU as the selection of resistant cells in this manner is thought to be more realistic. In the present study, the cellular concentrations of ACNU in C6/ACNU cells were almost 4 times lower than those found in C6 cells. Reserpine has enhanced the cytotoxicity of ACNU in C6/ACNU cells, and could completely overcome ACNU resistance *in vitro*. At a nontoxic dose of 20 μM of reserpine, the cellular level of ACNU in C6/ACNU cells increased to almost the same extent as that in C6 cells (Figure 2). Actually, in *in vitro* experiments, the sensitivities of C6 and C6/ACNU cells to ACNU were almost equal when 10 μM reserpine was added along with ACNU to the culture (Figure 1, Table I).

While examining a number of membrane interacting agents, it was found that reserpine enhances the cytotoxicity of ACNU in ACNU-resistant glioma cells and increases the intracellular uptake of ACNU in the cells. The mechanism of enhancement of the cytotoxicity of ACNU in C6/ACNU cells by reserpine is not known. However, Koshiura *et al.* (1980) discovered the enhanced effect of the chemotherapeutic agent 1-(gamma-chloropropyl)-2-chloromethylpyrimidine hydrochloride (CAP-2), in AH-13 and AH-44 cells by reserpine and reported the mechanism to be inhibition of DNA repair. Contrary to this report, we showed an increase of cellular uptake of ACNU in both C6 and C6/ACNU cells treated with reserpine. It is suggested that the enhanced effect of the cytotoxicity of ACNU in C6/ACNU cells is due to the increase of intracellular ACNU. This is supported by Inaba *et al.* (1981) to the effect that reserpine circumvented resistance to adriamycin and vincristine in P388 leukaemia cells. As for the actions of reserpine, antitumour activity against hypoxic cells was also reported (Lehnert, 1982), but the precise mechanism of this activity has not been clarified. On the other hand, it has been disclosed that reserpine decreases the amount of calcium in the heart, blood vessels and brain (Carrier *et al.*, 1970; Ross *et al.*, 1974), and that calcium bound to lipid of cell membranes is released by reserpine (Wang & Radouco-Thomas, 1978). Recently, Tsuruo *et al.* (1983) reported that the mechanism of drug resistance is due to both the reduced uptake of the drug and the increased active efflux of the intracellular drug from the resistant cells showing the intracellular accumulation of the drug by calcium antagonists. They have stressed that the mechanism of drug resistance is profoundly related to cell membrane calcium metabolism and calmodulin. The results of the present experiment are consistent with their observation of reduced cellular uptake of the drug in the resistant cells. Furthermore, the enhanced cytotoxicity of ACNU by reserpine could be explained by their hypothesis, if reserpine acts on the membrane as a calcium antagonist as reported previously. However, the data in the present study indicated an almost 50-fold reduction in the $IC_{50}$ (for 20 $\mu$M reserpine) in association with a four-fold increase in drug uptake. Although the enhanced effect of the cytotoxicity of ACNU could be partially explained by the increased cellular drug level, it is conceivable that other mechanisms also apply. Kokunai *et al.* (1985) reported that ACNU resistance in 9L glioma is due to the increased DNA repair. Aida *et al.* (1985) also referred to the increase of DNA repair in an ACNU-resistant human glioma cell line (HU-188) in which the activity of $O^6$-methyl guanine ($O^6$-mGua) DNA methyltransferase was high, and they reported that the increased activity of this enzyme is one of the causes of ACNU resistance in HU-188 human glioma. On these considerations, drug resistance is multifactorial and involves calcium, the enzymes related to calcium metabolism and cyclic AMP of the cell membranes (Inaba *et al.*, 1981) and DNA repair (Aida *et al.*, 1985; Kokunai *et al.*, 1985). Although the enhanced effect of the cytotoxicity of ACNU by reserpine presented herein might also be related to these factors, it has been shown that the mechanism of the enhanced effect of reserpine is partially due to the increase of the cellular accumulation of ACNU. The mechanism of ACNU resistance has not been elucidated. However, it seems that it is closely related to reduced uptake of ACNU as advocated in the present study. In order to investigate further the mechanism involved in ACNU resistance in C6/ACNU glioma, drug efflux in relation to reserpine should be measured.

It might be very difficult to explain the mechanism of drug resistance by a single mechanism as it is generally considered that the cause of drug resistance differs depending on the type of tumour and the drug. However, we are currently attempting to overcome ACNU resistance in rat brain tumour models using the same cell lines as those used in the present experiment. Moreover, we are investigating the exact basis of ACNU resistance by drug uptake and efflux studies in ACNU-resistant glioma cell lines.

Appreciation is extended to Dr William R. Shapiro for helpful comments during the preparation of this manuscript and to Dr Takao Hoshino for gifts of C6 glioma cells. We are also indebted to Mrs Rosalind Yoshida for editing the manuscript.

### References

AIDA, T., ABE, H. & BODELL, W.J. (1985). Mechanisms of cellular resistance to ACNU in human glioma cell lines. Abstracts of the 44th congress of the Japan Neurosurgical Society (Nagasaki). p. 156.

CARRIER, O. Jr., WHITTINGTON-COLEMAN, P.J., MATHENY, J. & SHIBATA, S. (1970). Tissue calcium losses following reserpine administration in rabbits. *Arch. Int. Pharmacodyn.*, **187**, 97.

INABA, M., FUJIKURA, R., TSUKAGOSHI, S. & SAKURAI, Y. (1981). Restored *in vitro* sensitivity of adriamycin- and vincristine-resistant P388 leukemia with reserpine. *Biochem Pharmacol.*, **30**, 2191.

KOKUNAI, T., SATANI, H., IZICHI, A., TAOMOTO, K., TAMAKI, N. & MATSUMOTO, S. (1985). Overcoming of ACNU-resistance of glioma cells with the use of inhibitors of poly (ADP-ribose) polymerase. Abstracts of the 44th congress of the Japan Neurological Society (Nagasaki). p. 56.

KOSHIURA, R., MIYAMOTO, K. & SANAE, F. (1980). Combination antitumor effect with central nervous system depressants on rat ascites hepatomas. *Gann.*, **71**, 45.

LEHNERT, S. (1982). Toxicity of diethylaminoresperine to a transplantable tumor: The significance of the presence of hypoxic cells. *Cancer Res.*, **42**, 3028.

MERRY, S., KAYE, S.B. & FRESHNEY, R.I. (1984). Cross-resistance to cytotoxic drugs in human glioma cell lines in culture. *Br. J. Cancer*, **50**, 831.

ROSS, D.H., MEDINA, M.A. & CARDENAS, H.L. (1974). Morphine and ethanol: Selective depletion of regional brain calcium. *Science*, **186**, 63.

SALMON, S.E., HAMBURGER, A.W., SOEHNLEN, B., DURIE, B.G.M., ALBERTS, D.S. & MOON, T.E. (1978). Quantitation of differential sensitivity of human-tumor stem cells to anticancer drugs. *N. Engl. J. Med.*, **298**, 1321.

SHIMIZU, K., USHIO, Y., HAYAKAWA, T. & MOGAMI, H. (1980). Combination chemotherapy with 1-(4-amino-2-methyl-5-pyrimidinyl) methyl-3-(2-chloroethyl)-3-nitrosourea hydrochloride and bleomycin in meningeal carcinomatosis in rats. *Cancer Res.*, **40**, 1341.

TSURUO, T., IIDA, H., TSUKAGOSHI, S. & SAKURAI, Y. (1983). Potentiation of vincristine and adriamycin effects in human hemopoietic tumor cell lines by calcium antagonists and calmodulin inhibitors. *Cancer Res.*, **43**, 2267.

USHIO, Y., SHIMIZU, K., ARAGAKI, Y., ARITA, N., HAYAKAWA, T. & MOGAMI, H. (1981). Alteration of blood-CSF barrier by tumor invasion into the meninges. *J. Neurosurg.*, **55**, 445.

WANG, Y.K. & RADOUCO-THOMAS, S. (1978). Calcium-reserpine interactions studied by electron spin resonance spectroscopy in spin labelled artificial membranes. *Prog. Neuro-Psychopharmac.*, **2**, 87.

YOSHIDA, T., USHIO, Y., HAYAKAWA, T. & 4 others. (1984). Development of ACNU-resistant Meningeal Gliomatosis Models: Establishment of resistant rat glioma sublines against ACNU. *Neurol. Surg.*, **12**, 1029.